# Long noncoding RNA *SLC2A1‐AS1* regulates aerobic glycolysis and progression in hepatocellular carcinoma via inhibiting the STAT3/FOXM1/GLUT1 pathway

**DOI:** 10.1002/1878-0261.12666

**Published:** 2020-03-30

**Authors:** Runze Shang, Miao Wang, Bin Dai, Jianbing Du, Jianlin Wang, Zekun Liu, Shibin Qu, Xisheng Yang, Jingjing Liu, Congcong Xia, Lin Wang, Desheng Wang, Yu Li

**Affiliations:** ^1^ Department of Hepatobiliary Surgery Xijing Hospital Fourth Military Medical University (Air Force Medical University) Xi'an China; ^2^ State Key Laboratory of Cancer Biology Cell Engineering Research Center & Department of Cell Biology Fourth Military Medical University (Air Force Medical University) Xi'an China; ^3^ Department of General Surgery General Hospital of the Central Theater Command of the People's Liberation Army Wuhan China; ^4^ School of Life Science Northwestern Polytechnical University Xi'an China

**Keywords:** FOXM1, GLUT1, glycolysis, hepatocellular carcinoma, long noncoding RNA, STAT3

## Abstract

Hepatocellular carcinoma (HCC) is one of the most lethal malignant diseases worldwide. Despite advances in the diagnosis and treatment of HCC, its overall prognosis remains poor. Recent studies have shown that long noncoding RNAs (lncRNAs) play crucial roles in various pathophysiological processes, including liver cancer. In the current study, we report that lncRNA *SLC2A1‐AS1* is frequently downregulated in HCC samples, as shown by quantitative real‐time polymerase chain reaction analysis. *SLC2A1‐AS1* deletion is significantly associated with recurrence‐free survival in HCC. By performing glucose uptake, lactate production and ATP detection assays, we found that *SLC2A1‐AS1*‐mediated glucose transporter 1 (GLUT1) downregulation significantly suppressed glycolysis of HCC. *In vitro* Cell Counting Kit‐8, colony formation, transwell assays as well as *in vivo* tumorigenesis and metastasis assays showed that *SLC2A1‐AS1* overexpression significantly suppressed proliferation and metastasis in HCC through the transcriptional inhibition of GLUT1. Results from fluorescence in situ hybridization, ChIP and luciferase reporter assays demonstrated that *SLC2A1‐AS1* exerts its regulatory role on GLUT1 by competitively binding to transketolase and signal transducer and activator of transcription 3 (STAT3) and inhibits the transactivation of Forkhead box M1 (FOXM1) via STAT3, thus resulting in inactivation of the FOXM1/GLUT1 axis in HCC cells. Our findings will be helpful for understanding the function and mechanism of lncRNA in HCC. These data also highlight the crucial role of *SLC2A1‐AS1* in HCC aerobic glycolysis and progression and pave the way for further research regarding the potential of *SLC2A1‐AS1* as a valuable predictive biomarker for HCC recurrence.

## Graphical abstract

Long noncoding RNAs play crucial roles in various pathophysiological processes including liver cancer. In this study, we report that antisense lncRNA *SLC2A1‐AS1* is downregulated in HCC. Overexpression of *SLC2A1‐AS1* inhibits aerobic glycolysis and progression of HCC. Mechanistically, *SLC2A1‐AS1* interacts with transketolase and signal transducer and activator of transcription 3 and inhibits Forkhead box M1/glucose transporter 1 axis activation in HCC cells.

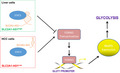

**Abbreviations**
FISH: fluorescence in situ hybridization
FOXM1: Forkhead box M1
GLUT1: glucose transporter 1
HCC: hepatocellular carcinoma
HE: haematoxylin and eosin
IHC: immunohistochemistry
lncRNAs: long noncoding RNAs
qRT‐PCR: quantitative real‐time polymerase chain reaction
STAT3: transketolase and signal transducer and activator of transcription 3

## 1 Introduction

Hepatocellular carcinoma (HCC) is the second leading cause of cancer‐related death worldwide (Armengol *et al.*, 2018). Despite advances in diagnosis and treatments, the overall prognosis for HCC remains extremely low due to the high rate of recurrence and distant metastasis (Xia *et al.*, 2013). Therefore, clarifying the underlying molecular mechanism of HCC progression is urgently needed to find effective therapeutic targets and recurrence predictors.

Metabolic reprogramming is a vital property of cancer cells. Unlike normal cells, cancer cells prefer to use the glycolysis pathway for metabolizing glucose upon enhanced glucose uptake and lactate production. This phenomenon, referred to as the Warburg effect, confers cancer cells with great advantages regarding tumour growth, apoptosis resistance, metastasis and immune escape (Gatenby *et al.*, 2006; Kroemer and Pouyssegur, 2008). These extensive connections between cancer metabolism and aggressive cancer features suggest that targeting metabolic pathways may be a promising effective method for treating cancer patients. Recent research demonstrated that cancer metabolism reprogramming can be regulated by both coding and noncoding genes via targeting glycolysis‐related transporters, enzymes and signalling pathways (Cairns *et al.*, 2011; Lin *et al.*, 2018; Yang *et al.*, 2014). A previous study and our recent publication demonstrated that a key glycolytic transporter, glucose transporter 1 (GLUT1), is specifically overexpressed in HCC and promotes HCC cell glycolysis and progression (Amann *et al.*, 2009; Shang *et al.*, 2017). We further revealed that GLUT1 is transactivated by the transcription factor Forkhead box M1 (FOXM1) in different cancer types (Shang *et al.*, 2017; Wang *et al.*, 2016). However, knowledge of how noncoding genes regulate GLUT1 expression is lacking.

Recently, long noncoding RNAs (lncRNAs), which are defined as a class of RNAs composed of more than 200 nucleotides and with no protein‐coding ability, have been identified to participate in numerous physiological and pathological processes (Sahu *et al.*, 2015). lncRNAs have been reported to be dysregulated in various types of cancers and play multiple roles in cancer cell proliferation, metastasis and metabolism. Increasing evidence indicates that a subtype of lncRNAs—antisense lncRNAs—play critical roles in cancer progression via regulating the expression of genes on the opposite strand with protein‐coding ability (Li *et al.*, 2016; Sun *et al.*, 2016). However, the role and specific regulatory mechanism of critical oncogene‐related antisense lncRNAs in HCC are largely unknown.

In this study, we reported the expression and function of a previously identified but unannotated antisense lncRNA, *SLC2A1‐AS1,* in HCC. We found that *SLC2A1‐AS1* was frequently downregulated in HCC tissues. *SLC2A1‐AS1* inhibited HCC cell aerobic glycolysis and progression through inactivating the key glycolytic transporter GLUT1, which is coded by the opposite strand coding gene *SLC2A1*. In detail, competitive binding of *SLC2A1‐AS1* to the transcriptional factor STAT3 inactivates the FOXM1/GLUT1 axis in HCC cells. To the best of our knowledge, our study is the first to investigate the role and mechanism of lncRNA *SLC2A1‐AS1* in HCC.

## 2 Materials and methods

### 2.1 Cell lines and culture conditions

The human nontumour liver cell line HL‐02 and human HCC cell lines MHCC97‐H, Huh7, HepG2 and Hep3B (obtained from the Cell Bank of the Type Culture Collection of the Chinese Academy of Sciences) were cultured in Dulbecco’s modified Eagle’s medium (DMEM; Gibco, Grand Island, NY, USA) supplemented with 10% FBS and 100 U·mL^−1^ penicillin/streptomycin (HyClone, Logan, UT, USA) at 37° C in a humidified 5% CO_2_ atmosphere.

### 2.2 Clinical samples

Thirty‐three paired HCC samples and surrounding nontumour tissues were obtained during surgical resection at Xijing Hospital, Fourth Military Medical University, from 2011 to 2015. None of the patients received radiotherapy or chemotherapy before resection. All samples were immediately frozen in liquid nitrogen after resection and further analysed by quantitative real‐time polymerase chain reaction (qRT‐PCR). The study was approved by the Ethics Committee of Xijing Hospital, informed written consent to participate in this study was obtained from each patient, and the study methodologies conformed to the standards set by the Declaration of Helsinki.

### 2.3 RNA extraction and qRT‐PCR

Total RNA was extracted from cells or tissues using TRIzol reagent (Invitrogen Inc., San Diego, CA, USA) and converted to cDNA with a PrimeScript RT Reagent Kit (TaKaRa, Tokyo, Japan). qRT‐PCR was performed with SYBR Green Premix Ex Taq (TaKaRa) and detected using an iQ‐5 Real‐Time PCR Detection System (Bio‐Rad, Hercules, CA, USA). All results were normalized to β‐actin. The primers for qRT‐PCR are listed in Table S1.

### 2.4 Western blot analysis

Whole cells and tissues were lysed in RIPA Lysis Buffer (Beyotime, Shanghai, China) with protease inhibitors. The extracted proteins were loaded onto SDS/PAGE gels for separation and then transferred to nitrocellulose membranes. The signal was visualized by a ChemiDoc™ XRS + System (Bio‐Rad). Primary antibody information is listed in Table S2.

### 2.5 Plasmids, siRNA and cell transfection

Full‐length *SLC2A1‐AS1* was constructed and inserted in the PEX‐3 vector (Genecreate, Wuhan, China). siRNAs and negative control siRNAs were synthesized by GenePharm (Sunnyvale, CA, USA). All plasmids and siRNAs were transfected using Lipofectamine 2000 (Invitrogen Inc.).

### 2.6 Glucose uptake, lactate production and ATP detection assays

Glucose, lactate and ATP assay kits (Nanjing Jiancheng) were used to measure the glucose uptake, lactate production and intracellular ATP levels of HCC cells according to the manufacturer’s instructions. All the results were normalized to the protein quantification values.

### 2.7 Cell proliferation and colony formation assays

A Cell Counting Kit‐8 (CCK‐8) assay was used for cell proliferation analysis. In brief, five replicate cell samples were seeded into 96‐well plates at a density of 2.0 × 10^3^ cells per well. At the indicated time points, 90 μL of culture medium containing 10% serum and 10 μL of CCK‐8 reagent were added to each well. Following incubation for 2 h at 37 °C, the absorbance was measured at 450 nm. For the colony formation assay, cells were seeded at a density of 300 cells/well in a 6‐well plate and cultured in 3 mL of DMEM supplemented with 10% FBS for 2 weeks. Then, the colonies were fixed in 95% EtOH and stained with a 4 g·L^−1^ crystal violet solution for counting.

### 2.8 Cell migration assays

Cell migration ability was analysed with cell culture chambers. Briefly, 3 × 10^4^ transfected HCC cells were serum‐starved and seeded onto the membrane (24‐well insert) in the upper chamber with serum‐free medium. After incubation for 24 h, the cells were fixed with methanol for 5 min and stained with a 0.1% crystal violet solution for 30 min. The invaded and migrated cells were counted at 200× magnification from five random fields of each filter.

### 2.9 Animal studies

The protocols for the animal studies were approved by the Animal Experiment Administration Committee of the Fourth Military Medical University. Four‐week‐old male BALB/C nude mice were used for the animal studies. For tumourigenesis assays, transfected MHCC97‐H cells (1 × 10^6^) were injected subcutaneously into the flanks of nude mice, with five mice per group. Tumours were harvested and weighed at 4 weeks after injection. For the metastasis assays, transfected MHCC97‐H‐luc cells (2 × 10^6^) were injected through the tail vein of the mice. Thirty days later, the mice were anaesthetized and injected intraperitoneally with d‐luciferin (Caliper), and the bioluminescence was detected 15 min later. Then, the mice were euthanized, and the lungs were excised, fixed and paraffin‐embedded for haematoxylin and eosin (H&E) and immunohistochemistry (IHC) staining.

### 2.10 RNA fluorescence in situ hybridization (FISH)

Fluorescence‐labelled probes for *SLC2A1‐AS1*, 18S rRNA and U6 RNA were synthesized by Genecreate Company. The FISH assays were performed using a Ribo™ Fluorescent In Situ Hybridization Kit (RiboBio, Guangzhou, China) according to the manufacturer’s instructions, and images were acquired using an Olympus Fluoview laser scanning confocal microscope.

### 2.11 Luciferase reporter assay

Luciferase reporter assays were performed using a luciferase assay kit (Promega, Madison, WI, USA). The wild‐type and mutant GLUT1 promoters were cloned into a pGL3 basic vector and cotransfected with the Renilla luciferase reporter and *SLC2A1‐AS1* overexpression plasmids. The relative firefly luciferase activity was measured by a Dual Luciferase Assay System (Promega) at 48 h after transfection.

### 2.12 Chromatin immunoprecipitation (ChIP) assay

ChIP assays were conducted in Huh7 and MHCC97‐H cells as the manufacturers instructed (Millipore Corp., Billerica, MA, USA). A STAT3 antibody (Cell Signal Technology, Danvers, MA, USA) was used to perform the immunoprecipitation experiment. PCR primer sequences for the DNA fragments targeting the FOXM1 and GLUT1 promoters are listed in Table S3.

### 2.13 RNA immunoprecipitation (RIP) assay

RNA immunoprecipitation assays were performed using an EZ‐Magna RIP™ RNA‐Binding Protein Immunoprecipitation Kit (Millipore Corp.). Briefly, Huh7 cells were lysed and incubated with a STAT3 antibody (Cell Signal Technology) or normal mouse IgG (Millipore Corp.). Then, qRT‐PCR assays were conducted using the RNA fractions.

### 2.14 Statistical analysis

Statistical analyses were conducted using graphpad prism 5 software (GraphPad Software Inc., La Jolla, CA, USA). Representative data are shown as the mean ± SD. Two independent groups were analysed by two‐tailed Student’s *t*‐tests. The correlation between *SLC2A1‐AS1* and GLUT1 expression was analysed using Spearman's rank correlation. Survival curves were calculated using the Kaplan–Meier method and statistically compared using the log‐rank test. All technical repeats were performed at least three times.

## 3 Results

### 3.1 *SLC2A1‐AS1* is downregulated in HCC and inversely correlated with GLUT1 expression and HCC recurrence

**Fig. 1:**
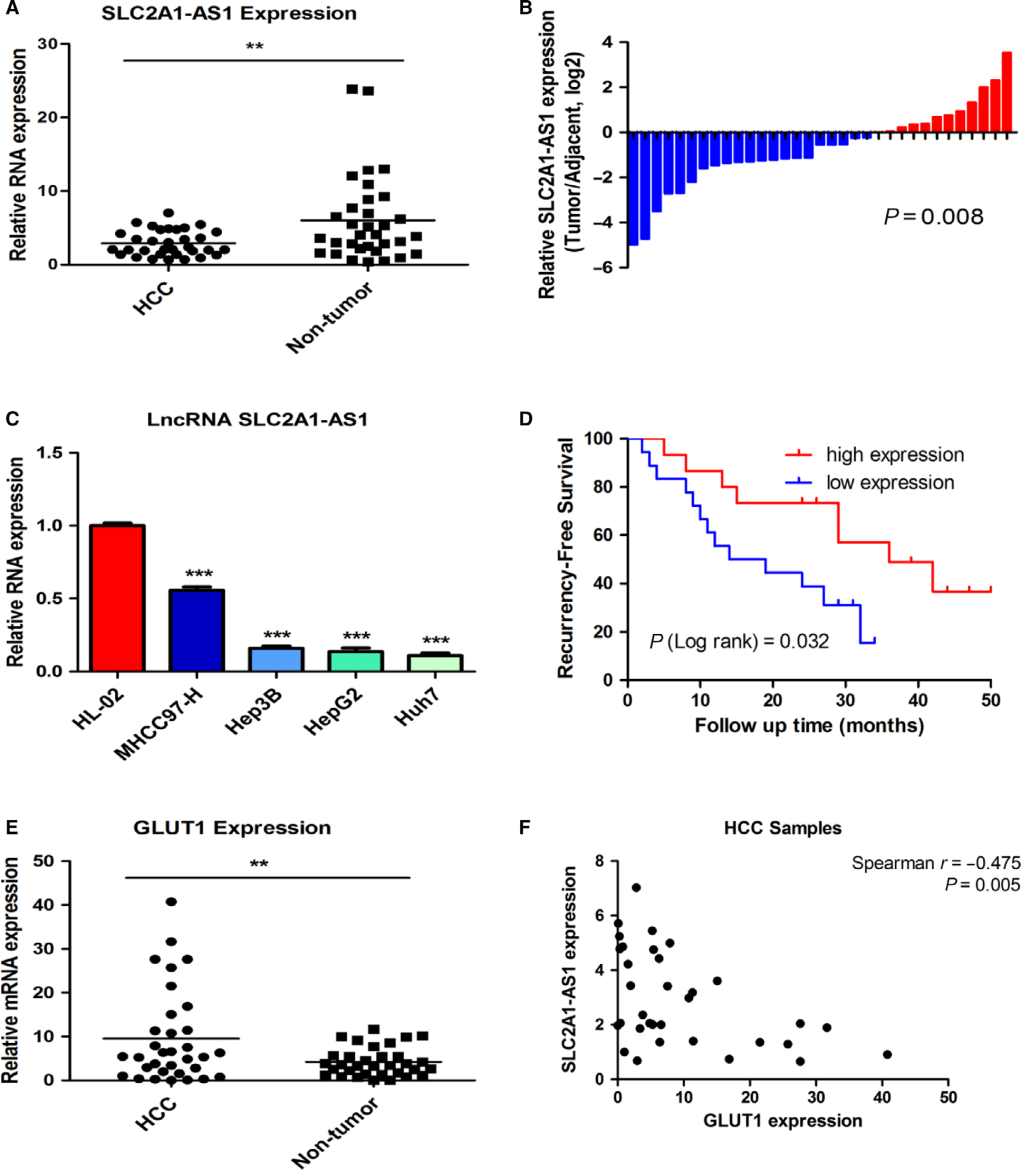
*SLC2A1‐AS1* is downregulated in HCC and correlated with tumour recurrence in HCC patients. (A, B) qRT‐PCR analysis of *SLC2A1‐AS1* expression levels in 33 HCC tissues and paired adjacent nontumour tissues. (C) Relative expression of *SLC2A1‐AS1* in normal liver cell lines and HCC cell lines according to qRT‐PCR analysis. (D) Kaplan–Meier curve for recurrence‐free survival based on *SLC2A1‐AS1* expression level. (E, F) qRT‐PCR analysis of GLUT1 mRNA expression in 33 HCC tissues and their paired adjacent nontumour tissues (E). The correlation of GLUT1 mRNA expression and *SLC2A1‐AS1* expression in HCC samples (F). Expression of *SLC2A1‐AS1* in HCC samples was analysed with Wilcoxon signed‐rank test; comparisons of the relative *SLC2A1‐AS1* levels between the groups were analysed with ANOVA followed by *post hoc* correction; Kaplan–Meier survival analysis was analysed with log‐rank test; the correlation between GLUT1 and *SLC2A1‐AS1* expression was analysed with Spearman's rank correlation. **P < 0.01, ***P < 0.001.

*SLC2A1‐AS1* is located at chromosomal band 1p34.2 (https://www.ncbi.nlm.nih.gov/; Fig. S1A), which has limited protein‐coding potential (https://lncipedia.org/; Fig. S1B). To investigate the expression levels of *SLC2A1‐AS1* in HCC, qRT‐PCR analysis was performed on a total of 33 HCC patient RNA samples that were extracted from both tumour samples and their matched adjacent nontumour counterparts. As shown in Fig. 1A,B, the expression of *SLC2A1‐AS1* was significantly downregulated in 63.6% (21/33) of the HCC samples compared with that in the paired nontumour samples. *In vitro*, we also found that *SLC2A1‐AS1* expression was lower in HCC cell lines than in normal liver cell line (Fig. 1C). Moreover, a Kaplan–Meier analysis indicated that patients with low *SLC2A1‐AS1* expression exhibited higher recurrence rates (Fig. 1D).

Recent investigations demonstrated that antisense transcripts may function as inhibitors or enhancers of corresponding gene expression. Therefore, we further assessed the mRNA expression of the coding gene *SLC2A1* (GLUT1), which is on the opposite strand of *SLC2A1‐AS1.* As expected, the mRNA expression of the oncogene *SLC2A1* was dramatically upregulated in HCC samples (Figs 1E and S2A) as well as in HCC cell lines (Fig. S2B). The Kaplan–Meier analysis indicated that patients with high GLUT1 mRNA expression exhibited higher recurrence rates (Fig. S2C). To evaluate whether *SLC2A1‐AS1* expression and GLUT1 expression were independently predictive of recurrence‐free survival in HCC patients, we performed multivariate Cox analysis. As shown in Table S4, *SLC2A1‐AS1* expression was an independent prognostic factor for recurrence‐free survival in patients with HCC.

Moreover, we observed a significant negative correlation between *SLC2A1‐AS1* expression and GLUT1 mRNA expression (Fig. 1F, *r *= −0.475, *P* = 0.005), which implies a potential regulation relationship between *SLC2A1‐AS1* and GLUT1.

Accordingly, these results suggested that *SLC2A1‐AS1* loss in HCC was correlated with HCC recurrence and inversely associated with GLUT1 expression.

### 3.2 *SLC2A1‐AS1* inhibits HCC glycolysis by negatively regulating GLUT1 expression

**Fig. 2:**
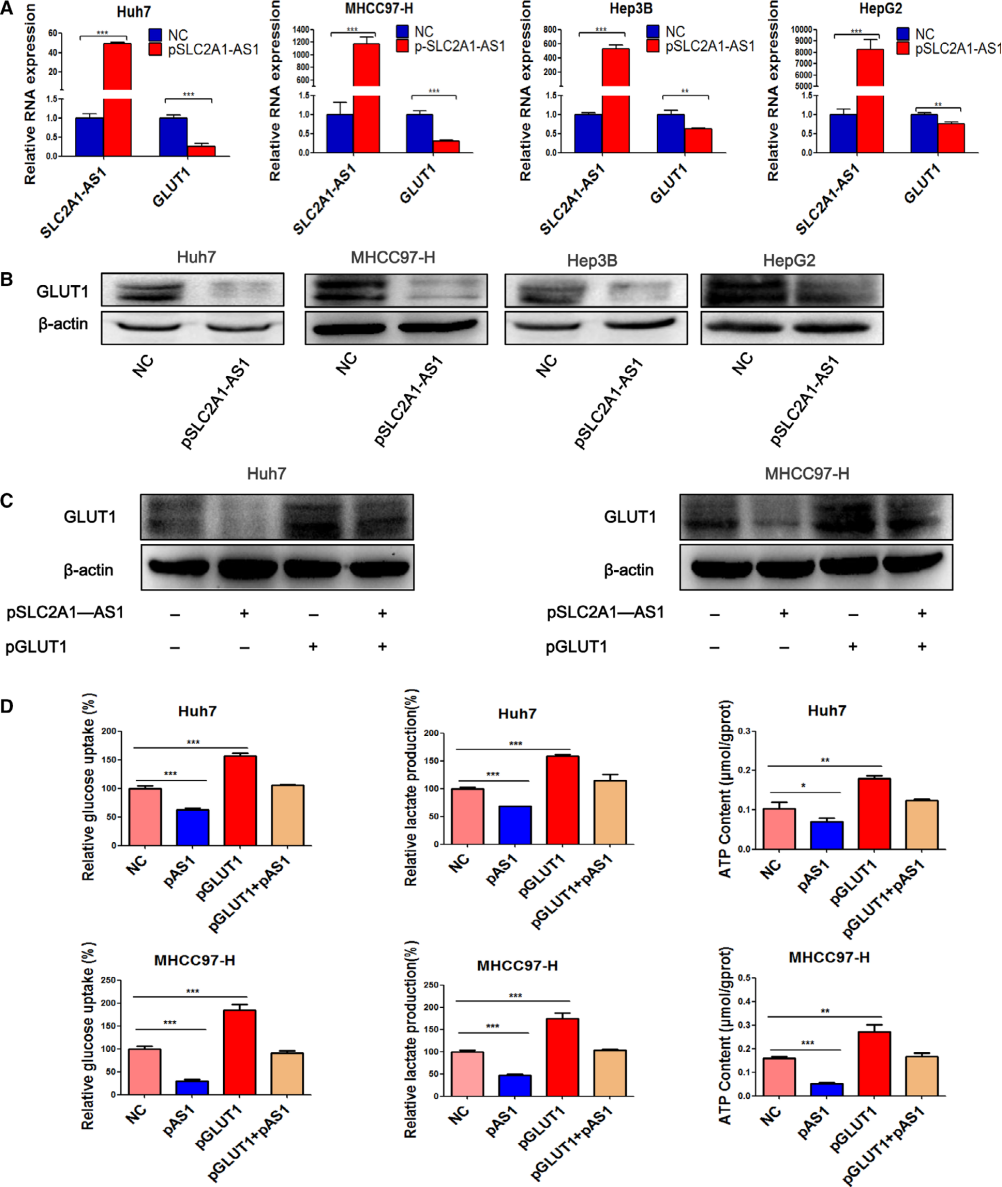
*SLC2A1‐AS1* regulates GLUT1 expression and HCC cell aerobic glycolysis. (A, B) Overexpression of *SLC2A1‐AS1* decreased the expression of GLUT1 at both the mRNA (A) and protein (B) levels in HCC cell lines. Data are expressed as mean ± SD of three independent experiments conducted in triplicate; they were analysed with Student’s *t*‐test. (C, D) Overexpression of *SLC2A1‐AS1* inhibits HCC cell glucose uptake, lactate production and intracellular ATP content, while overexpression of GLUT1 reversed these effects. Data are shown as means ± SD, and ANOVA followed by *post hoc* correction is used to calculate significance. pAS1, pSLC2A1‐AS1. *P < 0.05, **P < 0.01, ***P < 0.001.

To investigate whether *SLC2A1‐AS1* regulates GLUT1 expression, we induced *SLC2A1‐AS1* expression in HCC cell lines. We transfected the HCC cells with either 5 μg (Fig. 2A) or 0.5 μg (Fig. S3A) *SLC2A1‐AS1* overexpression plasmids for 24 h and then assessed the relative expression of *SLC2A1‐AS1* and GLUT1 mRNA. We found that *SLC2A1‐AS1* overexpression in varying degrees consistently reduced GLUT1 mRNA expression in four HCC cell lines (Figs 2A and S3A). Western blot results further confirmed the reduction in GLUT1 at the protein level after *SLC2A1‐AS1* transfection (Figs 2B and S3B). As a crucial glycolysis‐related transport protein, GLUT1 has been confirmed to be upregulated and participate in the activation of HCC aerobic glycolysis. Therefore, we investigated whether *SLC2A1‐AS1* could regulate HCC glycolysis by inhibiting GLUT1 expression. We transfected Huh7 and MHCC97‐H cells with *SLC2A1‐AS1* and GLUT1 plasmids alone or in combination and then detected GLUT1 protein expression (Fig. 2C). Moreover, we evaluated glycolytic activity by measuring glucose uptake, lactate production and intracellular ATP content. Notably, *SLC2A1‐AS1* overexpression prominently suppressed glucose uptake and lactate production and decreased intracellular ATP content in the two HCC cell lines (Fig. 2D). Intriguingly, GLUT1 upregulation reversed the effect of *SLC2A1‐AS1* overexpression in HCC glycolysis (Fig. 2D). These findings confirmed that *SLC2A1‐AS1* inhibited HCC glycolysis by negatively regulating GLUT1 expression.

### 3.3 *SLC2A1‐AS1* suppresses HCC cell proliferation and metastasis *in vitro* and *in vivo*

**Fig. 3:**
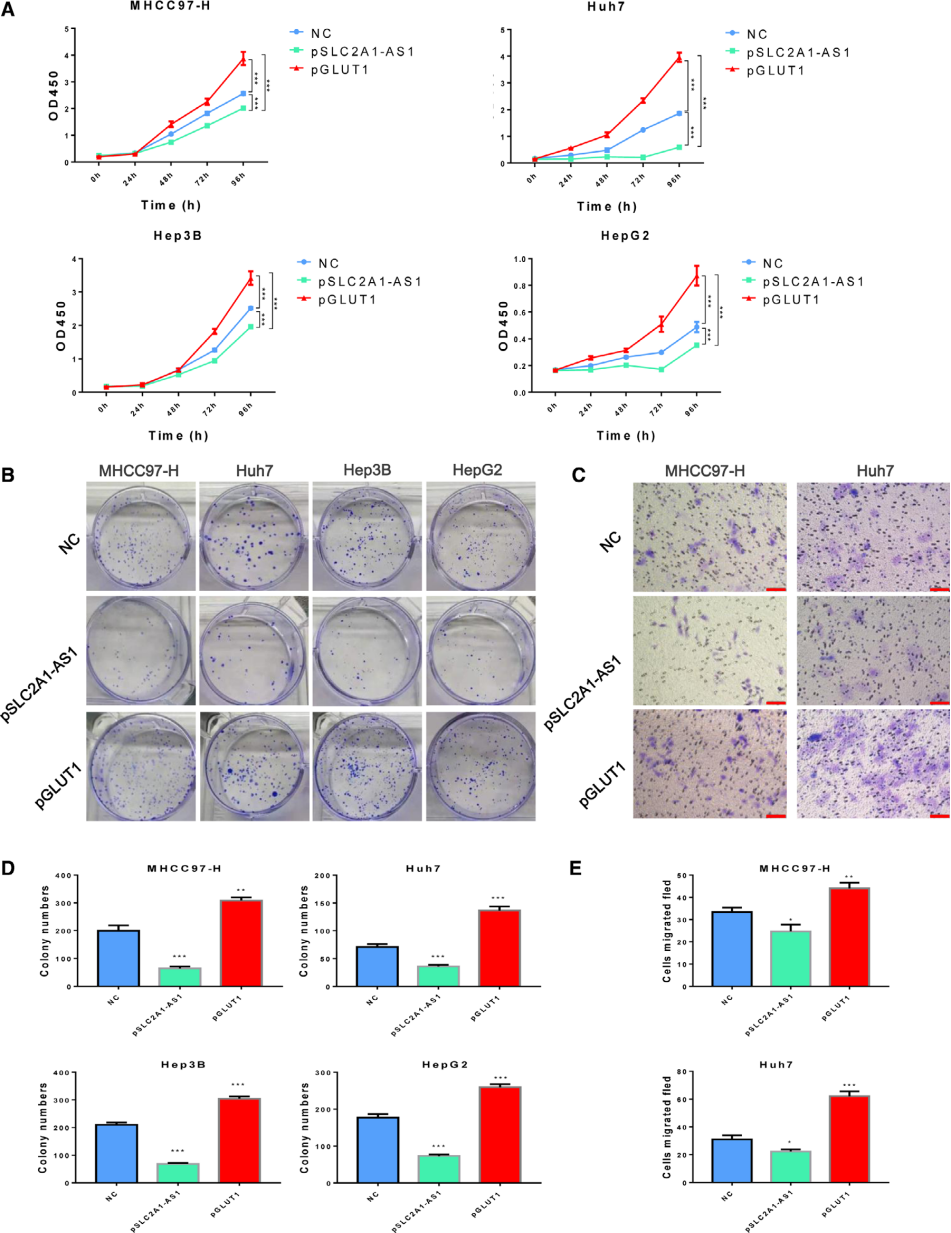
*SLC2A1‐AS1* inhibits HCC cell proliferation and metastasis *in vitro*. (A) HCC cell proliferation was assessed using CCK‐8 assays after *SLC2A1‐AS1* overexpression. (B) Overexpression of *SLC2A1‐AS1* significantly reduced colony formation in HCC cells. (C) Expression of *SLC2A1‐AS1* significantly reduced HCC cell migration. Scale bar = 100 μm. (D, E) Quantification of colonies (D) and migrated cells (E) with or without *SLC2A1‐AS1* overexpression. Data are shown as means ± SD, and ANOVA followed by *post hoc* correction is used to calculate significance. *P < 0.05, **P < 0.01, ***P < 0.001.

Metabolic reprogramming in cancer cells has been confirmed to contribute to tumour progression through a variety of mechanisms. Hence, we further explored whether *SLC2A1‐AS1* affects tumour progression accompanying metabolic changes. To determine the role of *SLC2A1‐AS1* in HCC cell proliferation *in vitro*, CCK‐8 and clone formation assays were performed in HCC cell lines after transfection with vector control plasmids or *SLC2A1‐AS1* overexpression plasmids. Meanwhile, the GLUT1 overexpression plasmid was also transfected in the same cells as positive control. As shown in Fig. 3A‐D, the proliferation ability was clearly reduced in MHCC97‐H, Huh7, Hep3B and HepG2 cells when *SLC2A1‐AS1* was overexpressed. In contrast, overexpression of GLUT1 accelerated proliferation in these cells. Transwell assays revealed that *SLC2A1‐AS1* suppressed the migration of HCC cells while GLUT1 induction promoted HCC migration. (Fig. 3C,E).

**Fig. 4:**
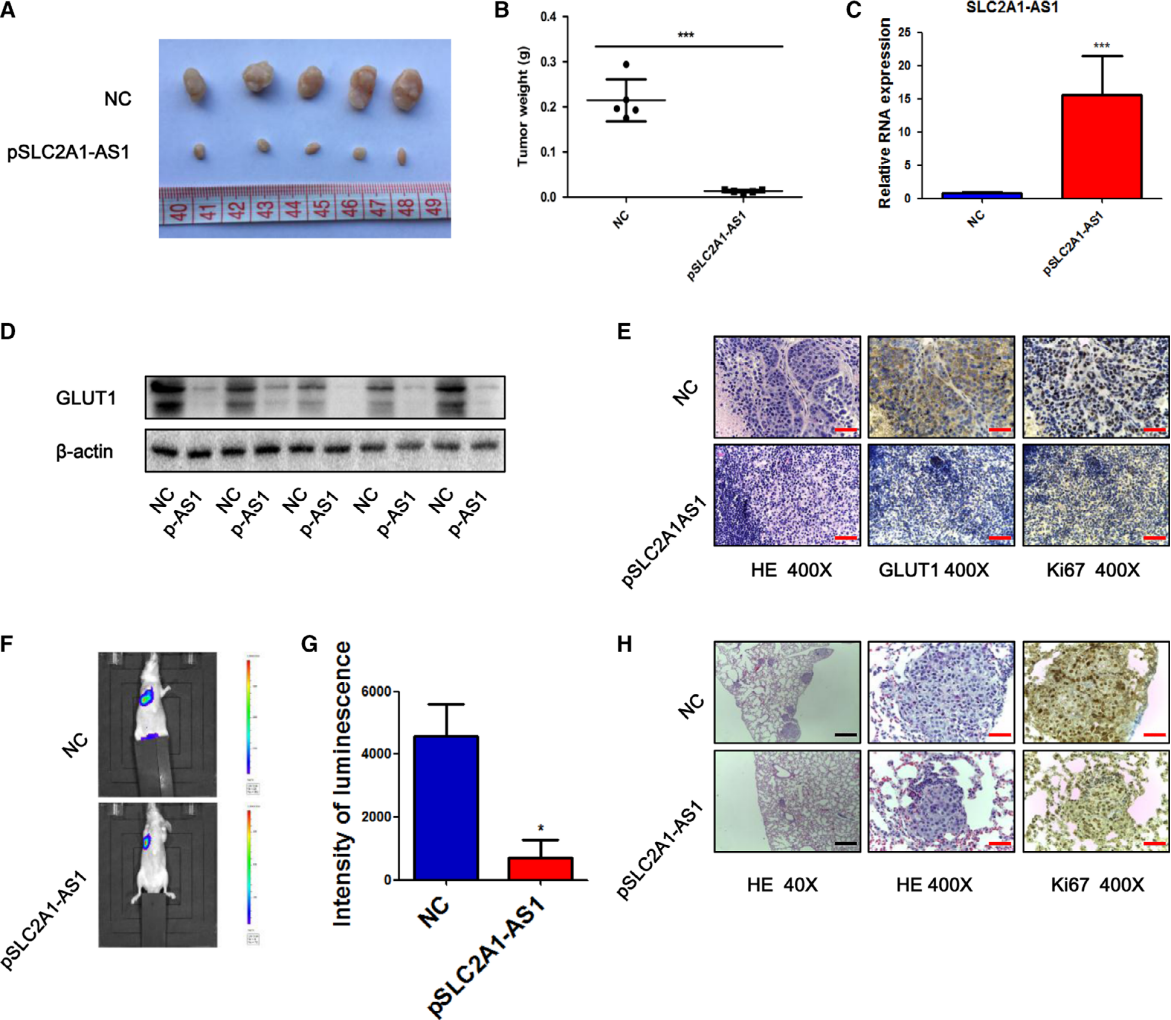
*SLC2A1‐AS1* inhibits tumour growth and metastasis *in vivo*. (A) Representative photograph of mice from each group injected with MHCC97‐H‐control or MHCC97‐H‐*SLC2A1‐AS1* cells. (B) Tumour weights were measured at day 28 in the control and *SLC2A1‐AS1* overexpression groups. (C) Representative *SLC2A1‐AS1* expression was analysed by qRT‐PCR. (D) Expression of GLUT1 was analysed by western blotting. (E) Representative images of H&E staining and immunohistochemical staining for Ki‐67 and GLUT1. Scale bar = 50 μm. (F, G) Overexpression of *SLC2A1‐AS1* significantly reduced the lung metastasis luminescence intensity. (H) Representative H&E and IHC staining for Ki‐67 in lung metastatic nodules are shown. Scale bar (black) = 500 μm, Scale bar (red) = 50 μm. pAS1, pSLC2A1‐AS1. Student’s *t*‐test was performed for statistical comparisons. *P < 0.05, ***P < 0.001.

To further validate the impact of *SLC2A1‐AS1* on tumour proliferation *in vivo*, MHCC97‐H cells overexpressing *SLC2A1‐AS1* were injected into the flanks of immunodeficient BALB/C nude mice to generate a subcutaneous xenotransplantation model. We observed that mice in the *SLC2A1‐AS1* overexpression group developed smaller tumours than mice in the control group (Fig. 4A,B). The overexpression of *SLC2A1‐AS1* in cell line‐derived tumour mass was confirmed by qRT‐PCR analysis (Fig. 4C). Subsequently, western blot and immunohistochemical staining showed that GLUT1 expression was significantly downregulated in the *SLC2A1‐AS1* overexpression group (Fig. 4D,E). Additionally, staining for the proliferation marker Ki67 was much weaker in the *SLC2A1‐AS1* overexpression group than in the NC group (Fig. 4E).

We also studied the effects of *SLC2A1‐AS1* expression on tumour metastasis *in vivo*. Luciferase‐labelled MHCC97‐H cells transfected with *SLC2A1‐AS1* or control plasmid were injected into nude mice via their tail vein. The bioluminescent signal was detected after the completion of 30 days postinjection. Representative bioluminescent images showed intense fluorescence in the NC group but very weak fluorescence in the *SLC2A1‐AS1* group (Fig. 4F,G). Histological analysis further confirmed that the number of metastatic lung nodules and cell proliferation ability were decreased in the *SLC2A1‐AS1‐*overexpressing group (Fig. 4H).

Altogether, these results confirmed that *SLC2A1‐AS1* inhibits HCC cell proliferation and metastasis *in vivo* as well as *in vitro*.

### 3.4 Overexpression of *SLC2A1‐AS1* inhibits GLUT1 transcriptional activation

**Fig. 5:**
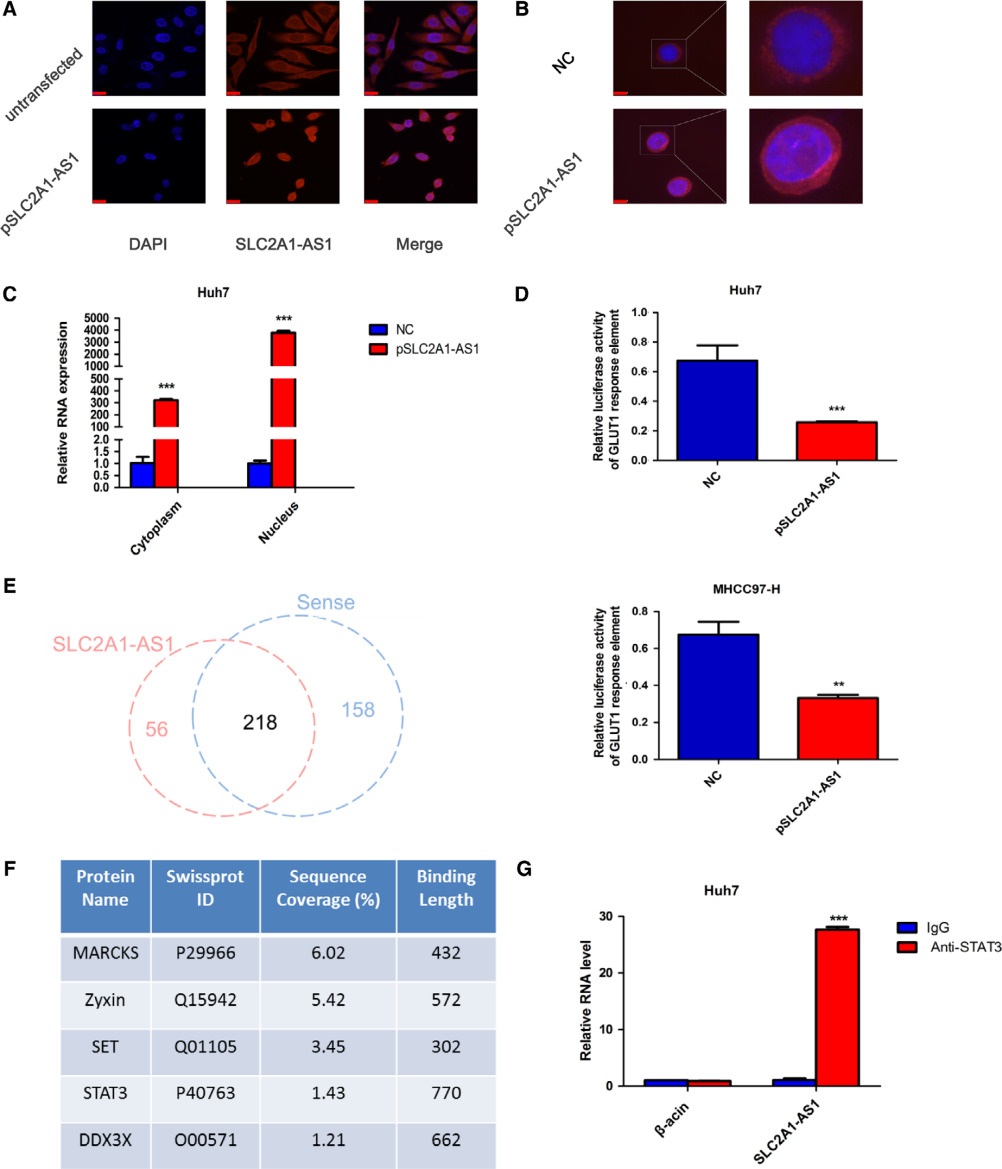
*SLC2A1‐AS1* regulates GLUT1 transcription and interacts with STAT3. (A) Subcellular distribution of *SLC2A1‐AS1* in Huh7 cells before and after *SLC2A1‐AS1* plasmid transfection by RNA FISH. Scale bar = 1900 μm. (B) Subcellular distribution of *SLC2A1‐AS1* in Huh7‐control and Huh7‐*SLC2A1‐AS1* cells by RNA FISH. Scale bar = 800 μm. (C) Cytoplasmic and nuclear fractions of Huh7 cells transfected with control or *SLC2A1‐AS1* plasmid were subjected to qRT‐PCR assays. (D) GLUT1 promoter activity in Huh7 and MHCC97‐H cells was assessed by luciferase reporter assays after *SLC2A1‐AS1* transfection. (E) Venn diagram showing *SLC2A1‐AS1‐*specific binding proteins from a biotin‐labelled RNA pulldown assay combined with mass spectrometric analysis. (F) *SLC2A1‐AS1*‐bound transcriptional factors identified by mass spectrometric analysis. (G) Cell lysates from Huh7 cells were immunoprecipitated with STAT3 or IgG antibody, and qRT‐PCR assays were subsequently performed to measure relative *SLC2A1‐AS1* expression. DAPI, 4’, 6‐diamidino‐2‐phenylindole. Student’s *t*‐test was performed for statistical comparisons. **P < 0.01, ***P < 0.001.

To study the mechanism of GLUT1 expression regulation by *SLC2A1‐AS1*, we first detected the subcellular distribution of *SLC2A1‐AS1* in HCC cells. The RNA FISH results revealed that *SLC2A1‐AS1* was localized mainly in the cytoplasm, but only very weak expression was shown (Fig. 5A). Intriguingly, a large amount of *SLC2A1‐AS1* accumulated in the cell nucleus when HCC cells were transfected with the *SLC2A1‐AS1* overexpression plasmid (Fig. 5A,B). This phenomenon was further confirmed by qRT‐PCR analysis of the nuclear/cytoplasmic RNA fractions (Fig. 5C). These results suggest that *SLC2A1‐AS1* could traffic through the nuclear envelope and may exert its function in the nucleus when overexpressed. Therefore, we next investigated whether *SLC2A1‐AS1* can regulate GLUT1 transcription. In this case, 1000 base pairs of the GLUT1 upstream promoter were cloned into the pGL3‐basic vector and used to perform a Dual‐Luciferase Reporter Assay. As shown in Fig. 5D, *SLC2A1‐AS1* overexpression significantly decreased the promoter activity of GLUT1 in both Huh7 and MHCC97‐H cells, suggesting an inhibitory role of *SLC2A1‐AS1* on GLUT1 transcription.

To further explore the specific mechanism of how *SLC2A1‐AS1* inhibits GLUT1 transcription, we first analysed the *SLC2A1‐AS1* sequence and found a potential ‘head‐to‐head’ pairing pattern composed of *SLC2A1‐AS1* and GLUT1 with 529 nucleotides with full complementarity (Fig. S1C). The ‘head‐to‐head’ or ‘head‐to‐tail’ complementary pairing pattern has been shown to play a crucial role in the regulation of antisense lncRNAs and their opposite strand coding genes (Chen *et al.*, 2018; Li *et al.*, 2012). Hence, we constructed two plasmids containing the complementary pairing sequence of *SLC2A1‐AS1* (pSLC2A1‐AS1‐part1) and the noncomplementary sequence of *SLC2A1‐AS1* (pSLC2A1‐AS1‐part2) separately for cell transfection. Unexpectedly, the qRT‐PCR results revealed that overexpression of pSLC2A1‐AS1‐part2, but not only pSLC2A1‐AS1‐part1, also reduced GLUT1 mRNA expression (Fig. S1D). Moreover, the reduction in GLUT1 in the pSLC2A1‐AS1‐part1‐transfected group was no more than that in the pSLC2A1‐AS1‐part2 overexpression group. These results indicate that ‘head‐to‐head’ pairing is not the principal regulatory mechanism of *SLC2A1‐AS1* and GLUT1.

### 3.5 *SLC2A1‐AS1* interacts with the transcriptional factor STAT3

To further explore the major regulatory mechanism by which *SLC2A1‐AS1* affects GLUT1 transcriptional activity, we focused on the transcriptional factors that interact with *SLC2A1‐AS1*. We performed a biotin‐labelled RNA pulldown assay and then subjected the precipitants to mass spectrometric analysis to identify proteins that interact with *SLC2A1‐AS1* in HCC cells. We found that 56 proteins interact with *SLC2A1‐AS1* uniquely (Fig. 5E and Table S5). Among these proteins, we screened five potential transcriptional factors (Fig. 5F). We focused particularly on STAT3, which has been demonstrated to be a key regulator of glucose metabolism in HCC (Li *et al.*, 2017; Liu and Yu, 2018; Wang *et al.*, 2012). To further validate the interactions between *SLC2A1‐AS1* and STAT3, we conducted an RNA immunoprecipitation assay and subsequent qRT‐PCR analysis. The results from these assays confirmed the enrichment of *SLC2A1‐AS1* in the STAT3 complex when compared with IgG (Fig. 5G).

### 3.6 *SLC2A1‐AS1*/STAT3 interaction inhibits the activity of the FOXM1/GLUT1 axis

The underlying regulatory mechanism of the *SLC2A1‐AS1*/STAT3 interaction in GLUT1 transcription was further examined. First, we explored whether the *SLC2A1‐AS1*/STAT3 interaction directly inhibits the transcriptional activation of GLUT1 via STAT3. Although STAT3 has been confirmed to participate in aerobic glycolysis in HCC, there is no evidence demonstrating that GLUT1 is a direct target of STAT3. Therefore, we first analysed the sequence of the GLUT1 promoter to identify potential STAT3‐binding sites. The bioinformatics analysis results revealed three potential STAT3‐binding sites present in the GLUT1 promoter (Fig. S4A). However, the ChIP assay results showed that GLUT1 chromatin did not specifically immunoprecipitate with antibodies against STAT3 when compared to IgG (Fig. S4B).

**Fig. 6:**
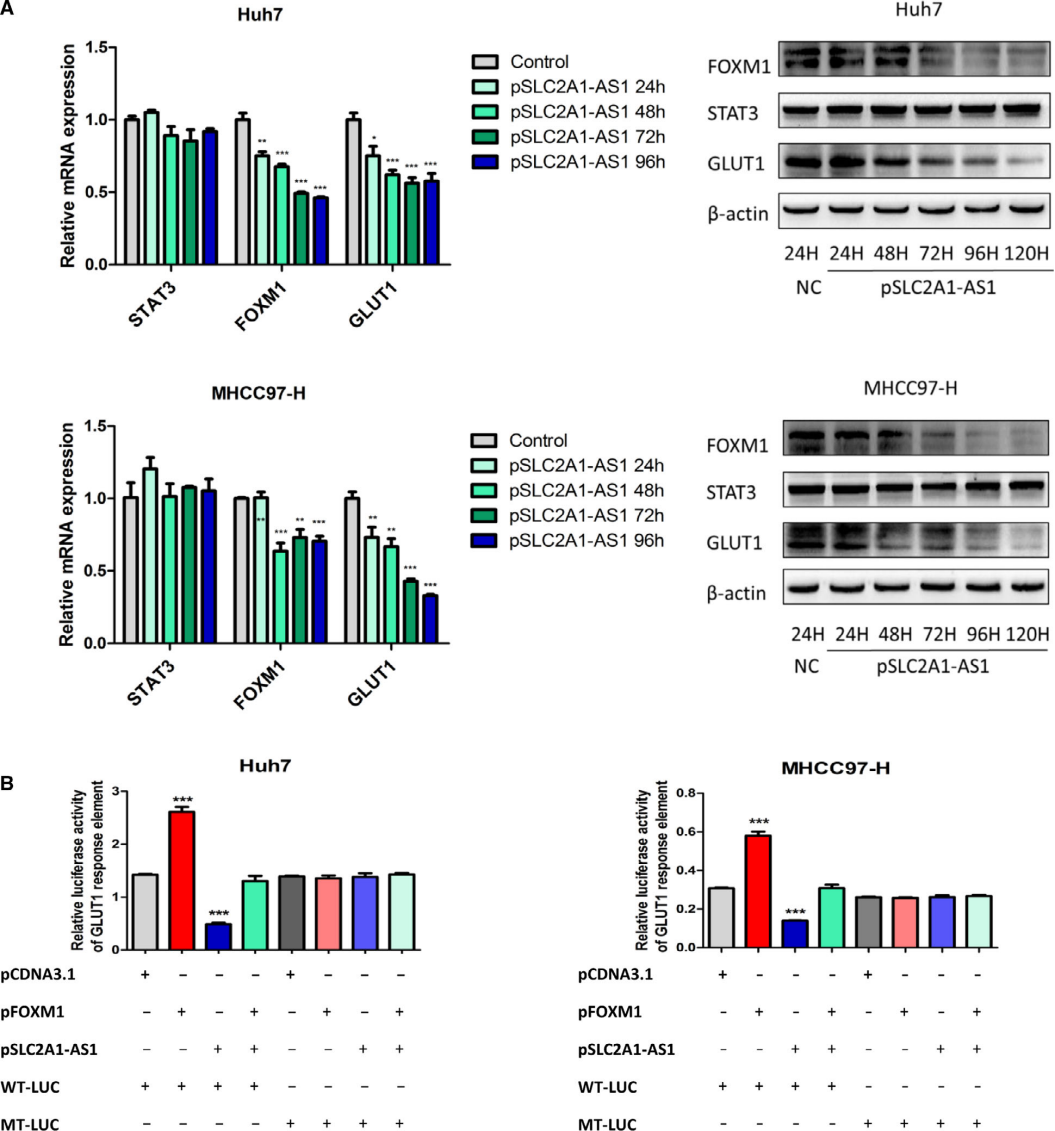
FOXM1 mediates the transcriptional regulation of GLUT1 via *SLC2A1‐AS1/*STAT3 interaction. (A) mRNA and protein expression levels of STAT3, FOXM1 and GLUT1 were assessed 24–96 h or 24–120 h after *SLC2A1‐AS1* plasmid transfection. (B) Reporter plasmids containing wild‐type (WT‐LUC) or mutant (MT‐LUC) FOXM1‐binding sites in the GLUT1 promoter were transfected in combination with control plasmid, FOXM1 overexpression plasmid, *SLC2A1‐AS1* overexpression plasmid or combined FOXM1 and *SLC2A1‐AS1* plasmids into Huh7 and MHCC97‐H cells. Relative luciferase activity was detected using a luciferase assay kit. Data were analysed with ANOVA followed by *post hoc* correction. *P < 0.05, **P < 0.01, ***P < 0.001.

Next, we explored whether the *SLC2A1‐AS1*/STAT3 interaction inhibits GLUT1 transcription via an indirect mechanism. The transcription factor FOXM1 has been demonstrated as a direct target of STAT3 (Mencalha *et al.*, 2012). Previously, we found that FOXM1 regulates the transcriptional activation of GLUT1 in HCC and ovarian cancer (Shang *et al.*, 2017; Wang *et al.*, 2016). Therefore, we hypothesized that FOXM1 mediates the regulation of GLUT1 via the *SLC2A1‐AS1*/STAT3 interaction. To verify our hypothesis, we first assessed whether the *SLC2A1‐AS1*/STAT3 interaction altered FOXM1 expression. As shown in Fig. 6A, HCC cells transfected with *SLC2A1‐AS1* showed a significant decrease in FOXM1 expression at both the mRNA and protein levels. However, the expression of STAT3 did not change at different time points after transfection. Moreover, the trend of altered GLUT1 expression at different time points after *SLC2A1‐AS1* transfection was consistent with the FOXM1 expression levels. Next, we cloned the sequence containing the FOXM1‐binding site in the GLUT1 promoter (ACAAATAA) or the corresponding mutant sequence (AtAcgatc) into the pGL3‐basic vector for transfection and performed luciferase reporter assays. We found that FOXM1 overexpression significantly increased wild‐type GLUT1 reporter activity, and this effect was reversed when *SLC2A1‐AS1* was overexpressed (Fig. 6B). Notably, these effects were abolished with the introduction of the mutant reporter construct (Fig. 6B). These results suggest that FOXM1 mediates the regulation of GLUT1 transcription via the *SLC2A1‐AS1*/STAT3 interaction.

**Fig. 7:**
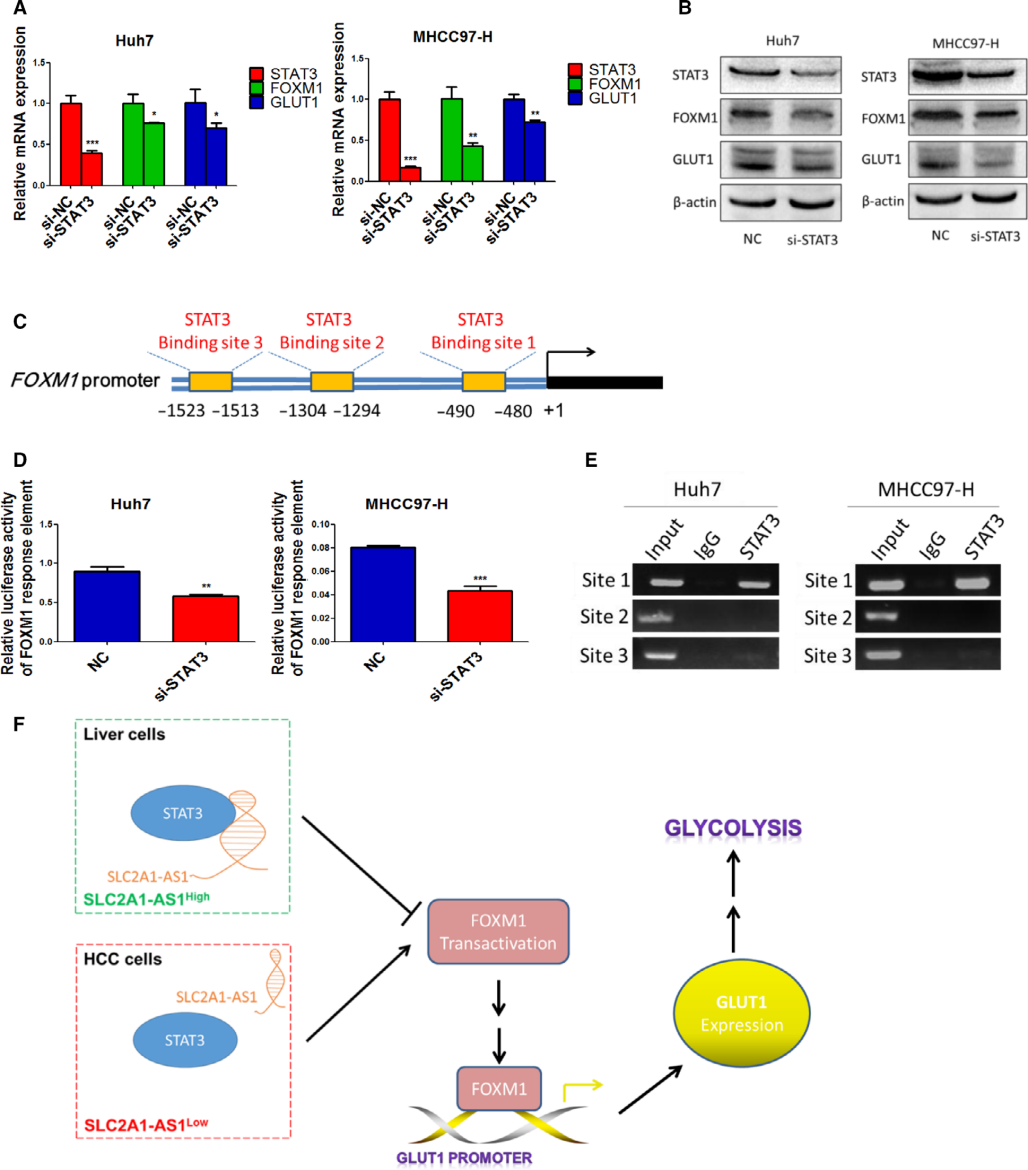
*SLC2A1‐AS1/*STAT3 interaction inhibits FOXM1 transcriptional activity and FOXM1/GLUT1 axis activation. (A, B) STAT3 knockdown significantly reduced the expression of FOXM1 and GLUT1 at the transcription (A) and translation (B) levels. (C) Putative STAT3‐binding sites in the FOXM1 promoter. (D) STAT3 knockdown significantly reduced the promoter activity of FOXM1. (E) ChIP analysis of the FOXM1 promoter using antibodies against STAT3 or IgG in Huh7 and MHCC97‐H cells. (F) A schematic model of the role and mechanism of *SLC2A1‐AS1* in HCC glycolysis. Student’s *t*‐test was performed for statistical comparisons. *P < 0.05, **P < 0.01, ***P < 0.001.

To further validate FOXM1 as a direct target of STAT3 in HCC, we transfected HCC cells with siRNA targeting STAT3 for further experiments. The qRT‐PCR and western blot assay results indicated that STAT3 knockdown significantly reduced both FOXM1 and GLUT1 expression (Fig. 7A,B). Moreover, downregulation of STAT3 significantly decreased the activity of the FOXM1 promoter (Fig. 7D). In addition, the ChIP assay results suggested that the region from −480 to −490 of the FOXM1 promoter is a crucial response element of STAT3 in HCC cells (Fig. 7C,E).

Next, to confirm whether FOXM1 is required for the *SLC2a1‐AS1*‐mediated GLUT1 downregulation, we conducted cell transfection with control plasmids and SLC2A1‐AS1 overexpression plasmids, or cotransfected with SLC2A1‐AS1 and FOXM1 overexpression plasmids in HCC cells. As shown in Fig. S5, overexpression of FOXM1 abrogated the SLC2a1‐AS1‐mediated GLUT1 downregulation without altering SLC2A1‐AS1 expression.

In summary, the above results indicate that the *SLC2A1‐AS1*/STAT3 interaction inhibits the transcriptional activation of FOXM1 via STAT3 and then reduces the activity of the FOXM1/GLUT1 axis.

## 4 Discussion

It has been well established that lncRNAs can affect coding gene expression through several mechanisms, including pretranscriptional, transcriptional and post‐transcriptional processing. Various studies have uncovered the crucial roles of lncRNAs in regulating carcinogenesis and progression. However, the roles of lncRNAs in tumour metabolism remain obscure. Specifically, the involvement of lncRNAs in HCC glycolysis and progression is not widely studied. In this study, we identified a previously unstudied lncRNA, *SLC2A1‐AS1* (Ensembl ID: ENSG00000227533), that is frequently downregulated in HCC cell lines and tissues and associated with recurrence‐free survival. This molecule acts as an antisense RNA of GLUT1. We further validated that *SLC2A1‐AS1* inhibits HCC cell glycolysis via negatively regulating GLUT1 expression. In addition, we observed that *SLC2A1‐AS1* overexpression markedly decreased the proliferation and metastasis of HCC cells. Moreover, we found *SLC2A1‐AS1* accumulation in the cell nucleus when it was overexpressed. We also found an interaction between *SLC2A1‐AS1* and STAT3, the latter of which is a transcription factor that plays crucial roles in tumour glycolysis. Furthermore, we determined that the *SLC2A1‐AS1*/STAT3 interaction weakens the activation of the FOXM1/GLUT1 axis.

Antisense lncRNA *SLC2A1‐AS1* is located on the opposite strand of *SLC2A1* (GLUT1) on chromosome 1. Antisense transcripts have been demonstrated to regulate the expression of corresponding sense‐strand mRNAs by affecting their transcription or mRNA stability (Nolasco *et al.*, 2012; Pan *et al.*, 2018). In the current study, we found that *SLC2A1‐AS1* inhibits the mRNA and protein expression of GLUT1, which indicates that *SLC2A1‐AS1* exerts its functional roles via regulating GLUT1 expression. The role of GLUT1 has been well established in cancer cells, especially in cancer metabolism. Cancer cells prefer to use the glycolysis pathway to meet their increased bioenergetics and biosynthetic demands, and they exhibit increased glucose uptake and lactate production. The glucose transporter (GLUT) family plays a vital role in glucose transport across the plasma membrane, which is the initial step of glycolysis. In different cancer cells, GLUT1, a member of the GLUT family, is frequently aberrantly expressed, which has potential effects on the glycolysis process in cancer (Szablewski, 2013). GLUT1 is abnormally overexpressed in HCC and promotes HCC cell glycolysis and progression (Amann *et al.*, 2009). Our results show that the decreased glycolysis ability in HCC cells caused by *SLC2A1‐AS1* overexpression can be reversed by GLUT1 overexpression. This observation indicates that *SLC2A1‐AS1* decreased HCC glycolysis through reducing GLUT1 expression.

As a vital connection between the tumour microenvironment and tumour progression, the process of tumour metabolic reprogramming has been regarded as cancer’s Achilles’ heel because this process contributes to tumour progression by promoting cell growth, invasion, metastasis, tumour angiogenesis and immunosurveillance (Kroemer and Pouyssegur, 2008). The primary benefit of glycolysis in tumour cells is that increased glucose levels provide a carbon source for anabolic reactions. Moreover, lactate, the principal end product of aerobic glycolysis, provides a favourable acidic microenvironment for tumour invasion and metastasis (Koukourakis *et al.*, 2006). Similarly, we found that *SLC2A1‐AS1* inhibits the production of lactate as well as glucose uptake in HCC cells. The results from *in vitro* and *in vivo* assays further demonstrate the inhibitory role of *SLC2A1‐AS1* in HCC proliferation and metastasis. These results indicate that *SLC2A1‐AS1* contributes to HCC glycolysis and then manipulates cell growth and migration abilities.

Although antisense lncRNAs have been shown to act at nearly every level of gene regulation, including pretranscription, transcription and post‐transcription (Villegas and Zaphiropoulos, 2015), the different subcellular localization of lncRNAs determines the specific regulatory molecular mechanism (Guttman and Rinn, 2012). lncRNAs accumulating in the cytoplasm commonly act as sponges for microRNAs and proteins and participate in post‐transcription regulation (Kim *et al.*, 2016; Liang *et al.*, 2015). However, in the nucleus, lncRNAs are involved in gene transcription (Zong *et al.*, 2011). *SLC2A1‐AS1* expression was relatively low in HCC cells and was located predominantly in the cytoplasm. However, we observed *SLC2A1‐AS1* accumulation in the cell nucleus when it was overexpressed. Hence, we speculated that *SLC2A1‐AS1* affected GLUT1 transcription. This hypothesis was validated by a luciferase reporter assay.

Recently, sense–antisense pairing was identified as an important mechanism of the regulation between antisense lncRNAs and their corresponding coding genes (Chen *et al.*, 2018; Hung and Chang, 2010; Li *et al.*, 2012; Morris *et al.*, 2008). We also found a ‘head‐to‐head’ pairing pattern between *SLC2A1‐AS1* and GLUT1. Nevertheless, we found that overexpression of both the pairing part and the nonpairing part of *SLC2A1‐AS1* reduced the mRNA expression of GLUT1. This indicates that ‘head‐to‐head’ pairing is not the principal regulatory mechanism of *SLC2A1‐AS1* and GLUT1.

Using a biotin‐labelled RNA pulldown assay and RNA immunoprecipitation assay, we revealed an interaction between *SLC2A1‐AS1* and the STAT3 protein. STAT3 is an important transcriptional factor that has been identified to regulate cancer initiation and progression (Ihle, 1996; Wang *et al.*, 2011). It has also been reported to play a role in cancer glycolysis (Li *et al.*, 2017; Li *et al.*, 2015; Wang *et al.*, 2013). We found that although *SLC2A1‐AS1* did not directly regulate the transcriptional activity of GLUT1, it inhibited the transcription of FOXM1, which we previously determined to be a crucial transcriptional activator of GLUT1 (Shang *et al.*, 2017; Wang *et al.*, 2016). These results therefore indicated a possible molecular link among *SLC2A1‐AS1*, STAT3 and the FOXM1/GLUT1 axis, but the precise binding site between *SLC2A1‐AS1* and STAT3 requires further study.

In summary, we report antisense lncRNA *SLC2A1‐AS1* as a tumour suppressor gene. *SLC2A1‐AS1* interacts with STAT3 and inhibits FOXM1/GLUT1 axis activation in HCC cells. We also characterized the suppressive effects that *SLC2A1‐AS1* exerts in glycolysis through inactivating GLUT1, which in turn inhibits HCC progression. Therefore, our study reveals the function and mechanism of *SLC2A1‐AS1* in HCC and highlights its prognostic and therapeutic significance for HCC patients.

## 5 Conclusion

*SLC2A1‐AS1* inhibits glycolysis and progression in HCC via the STAT3/FOXM1/GLUT1 axis. *SLC2A1‐AS1* can be considered a valuable predictive biomarker for HCC recurrence.

## Conflict of interest

The authors declare no conflict of interest.

## Author contributions

RS, MW and BD performed most experiments and analysed the data. RS wrote the manuscript. JD and JW performed prognosis and data analysis. ZL and SQ participated in the *in vitro* study. XY, JL participated in the *in vivo* study. CX collected HCC tissue samples and clinical data. YL, DW and LW designed the overall study, supervised the experiments and revised the paper.

## Supporting information


**Fig. S1** **.** Characterization of the antisense lncRNA *SLC2A1‐AS1*.
**Fig. S2**. GLUT1 is upregulated in HCC and associated with tumour recurrence in HCC patients.
**Fig. S3**.* SLC2A1‐AS1* inhibits GLUT1 expression.
**Fig. S4** **.** STAT3 cannot bind to the GLUT1 promoter in HCC cells.
**Fig. S5** **.** FOXM1 is required for *SLC2A1‐AS1*‐mediated GLUT1 downregulation.
**Table S1** **.** Primers used in qRT‐PCR assays.
**Table S2** **.** List of primary Antibodies.
**Table S3** **.** PCR primer sequences of DNA fragments targeted FOXM1 and GLUT1 promoters.
**Table S4** **.** Univariate and multivariate Cox regression analyses of recurrence‐free survival in 33 patients with HCC.
**Table S5** **.** Summary of proteins interacting with *SLC2A1‐AS1* uniquely by mass spectrometric analysis.
