# Supplementary material for: Long noncoding RNA SLC2A1‐AS1 regulates aerobic glycolysis and progression in hepatocellular carcinoma via inhibiting the STAT3/FOXM1/GLUT1 pathway
Source: Mol Oncol. 2020 Mar 30;14(6):1381–96. doi: 10.1002/1878-0261.12666 (PMC7266282; doi:10.1002/1878-0261.12666)
Supplement: Supplementary file 1 — Fig. S1 . Characterization of the antisense lncRNA SLC2A1‐AS1. Fig. S2. GLUT1 is upregulated in HCC and associated with tumour recurrence in HCC patients. Fig. S3. SLC2A1‐AS1 inhibits GLUT1 expression. Fig. S4 . STAT3 cannot bind to the GLUT1 promoter in HCC cells. Fig. S5 . FOXM1 is required for SLC2A1‐AS1‐mediated GLUT1 downregulation. Table S1 . Primers used in qRT‐PCR assays. Table S2 . List of primary Antibodies. Table S3 . PCR primer sequences of DNA fragments targeted FOXM1 and GLUT1 promoters. Table S4 . Univariate and multivariate Cox regression analyses of recurrence‐free survival in 33 patients with HCC. Table S5 . Summary of proteins interacting with SLC2A1‐AS1 uniquely by mass spectrometric analysis. [file MOL2-14-1381-s001.pdf]

Long noncoding RNA *SLC2A1-AS1* regulates aerobic glycolysis and progression in hepatocellular carcinoma via inhibiting the STAT3/FOXM1/GLUT1 pathway

Runze Shang#, Miao Wang#, Bin Dai#, Jianbing Du, Jianlin Wang, Zekun Liu, Shibin Qu, Xisheng Yang, Jingjing Liu, Congcong Xia, Lin Wang*, Desheng Wang*, Yu Li*

Table of contents

Fig. S1……………………………………………………………………………..………...2

Fig. S2……………………………………………………………………………..………...3

Fig. S3……………………………………………………………………………..………...4

Fig. S4……………………………………………………………………………..………...5

Fig. S5……………………………………………………………………………..………...6

Table S1…………………………………………………………………………...….......7

Table S2…………………………………………………………………………..……...8

Table S3…………………………………………………………………………..……...9

Table S4………………………………………………………..……………..……...10

Table S5………………………………………………………..……………..……...11

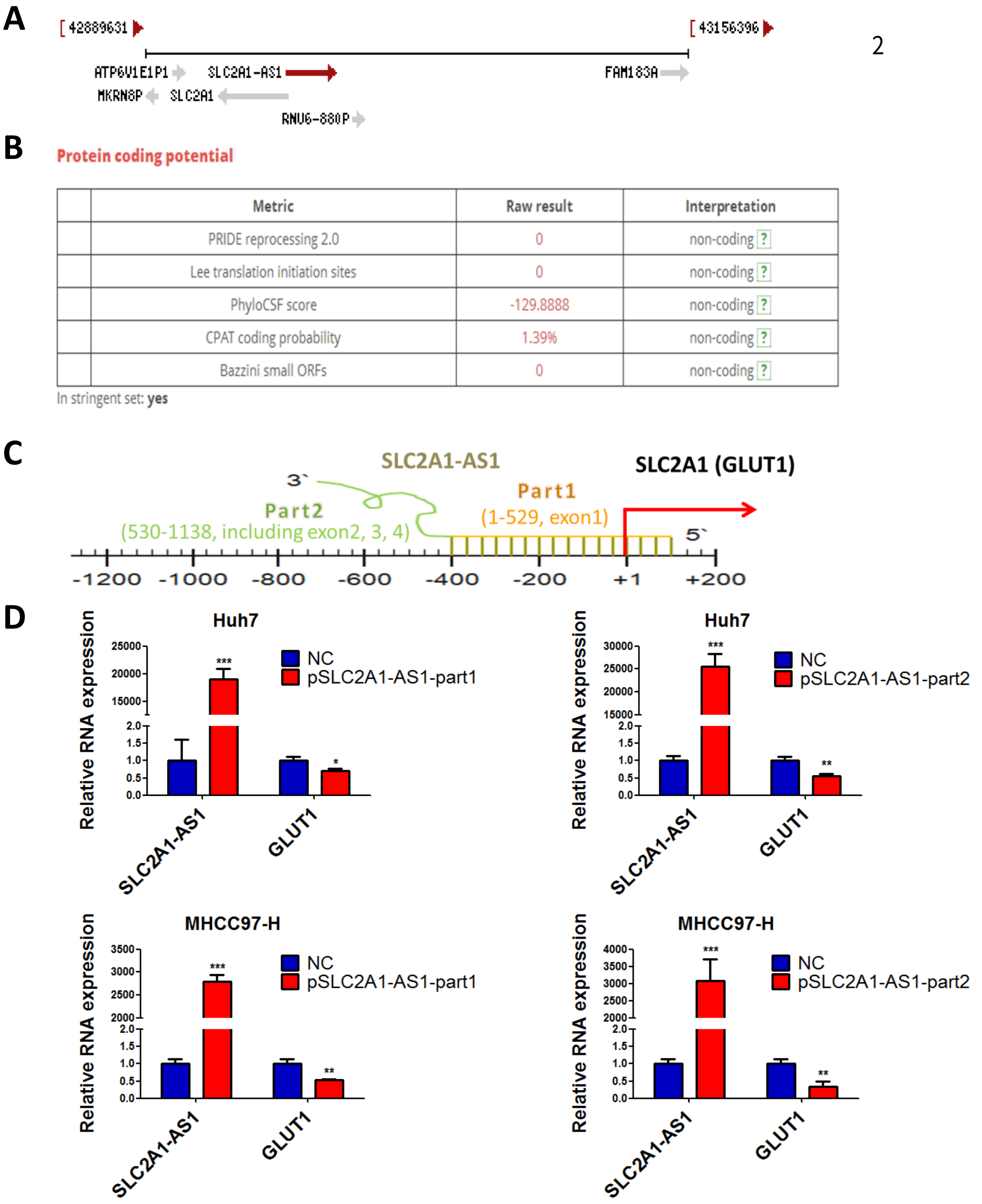

Fig. S1. Characterization of the antisense lncRNA *SLC2A1-AS1*. (**A**) Schematic diagram of the genomic locus of *SLC2A1-AS1* (https://www.ncbi.nlm.nih.gov/). (**B**) Prediction of the protein-coding potential of *SLC2A1-AS1* (https://lncipedia.org/). (**C**) Schematic representation of the potential overlap between *SLC2A1-AS1* and GLUT1. (**D**) qRT-PCR analysis was performed in HCC cells after transfection with one of the two parts of *SLC2A1-AS1.* Student's t-test was performed for statistical comparisons.*P<0.05, **P<0.01, ***P<0.001.

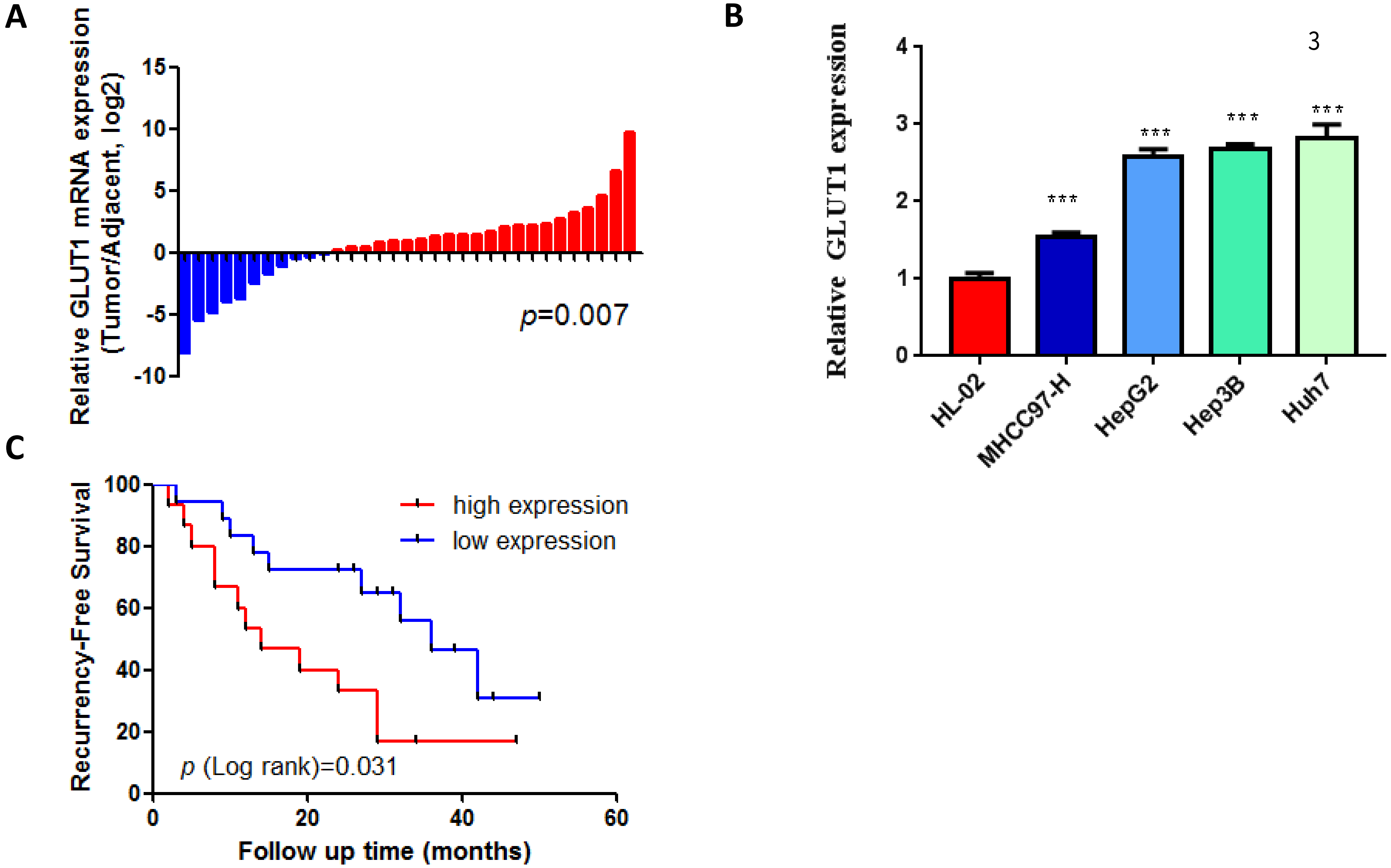

Fig. S2. GLUT1 is upregulated in HCC and associated with tumour recurrence in HCC patients. (**A**) qRT-PCR analysis of GLUT1 expression levels in 33 HCC tissues and paired adjacent non-tumour tissues. (**B**) Relative expression of GLUT1 in normal liver cell line and HCC cell lines according to qRT-PCR analysis. (**C**) Kaplan-Meier curve for recurrence-free survival based on GLUT1 mRNA expression level. Expression of GLUT1 in HCC samples was analysed with Wilcoxon signed-rank test; comparisons of the relative GLUT1 levels between the groups were analysed with ANOVA followed by post-hoc correction; Kaplan–Meier survival analysis was analysed with log-rank test; ****P*<0.001.

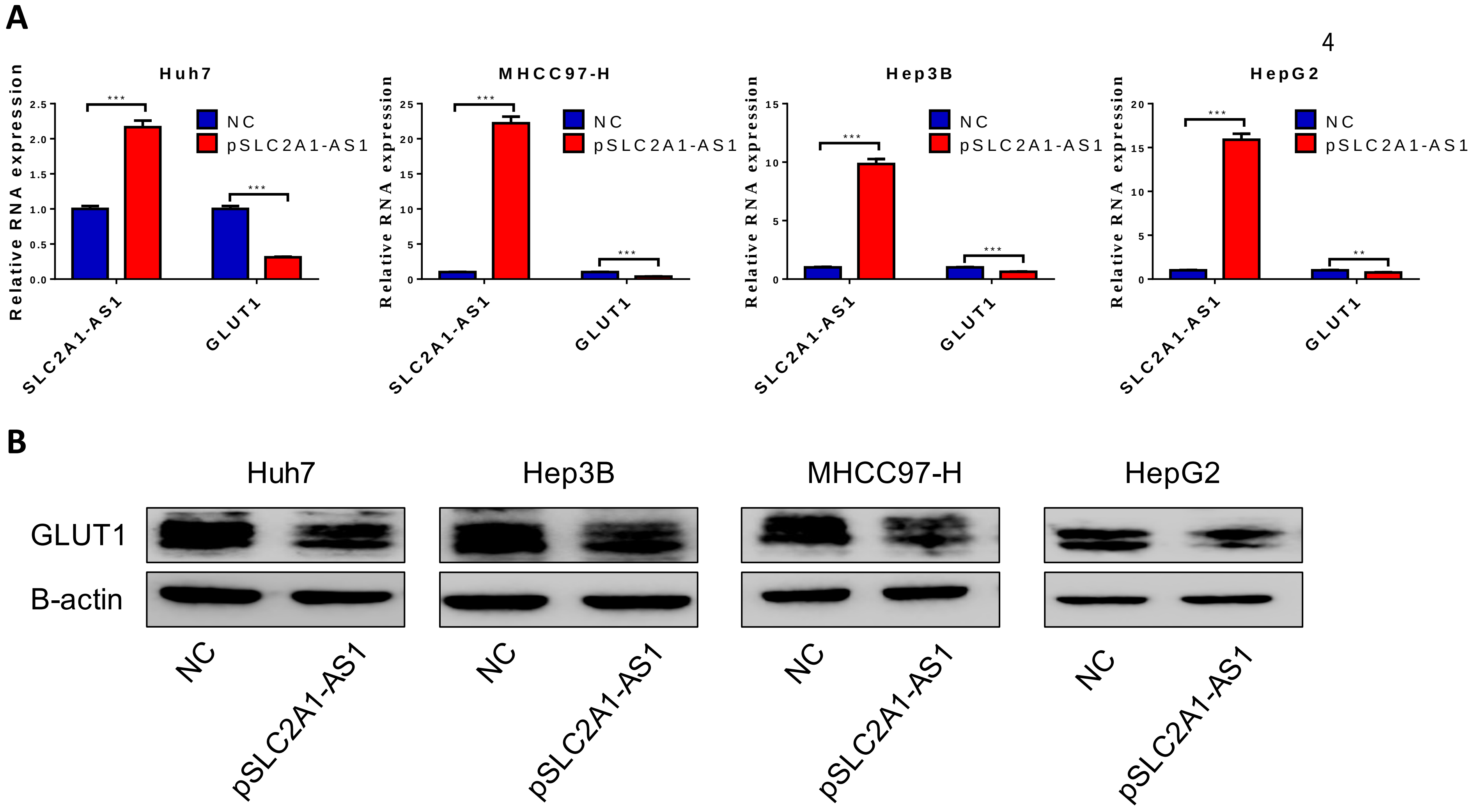

Fig. S3. *SLC2A1-AS1* inhibits GLUT1 expression. Overexpression of *SLC2A1-AS1* by transfecting 0.5 µg *SLC2A1-AS1* plasmids reduced both the mRNA (**A**) and protein (**B**) level of GLUT1 in HCC cell lines. Student's t-test was performed for statistical comparisons. ***P*<0.01, ****P*<0.001.

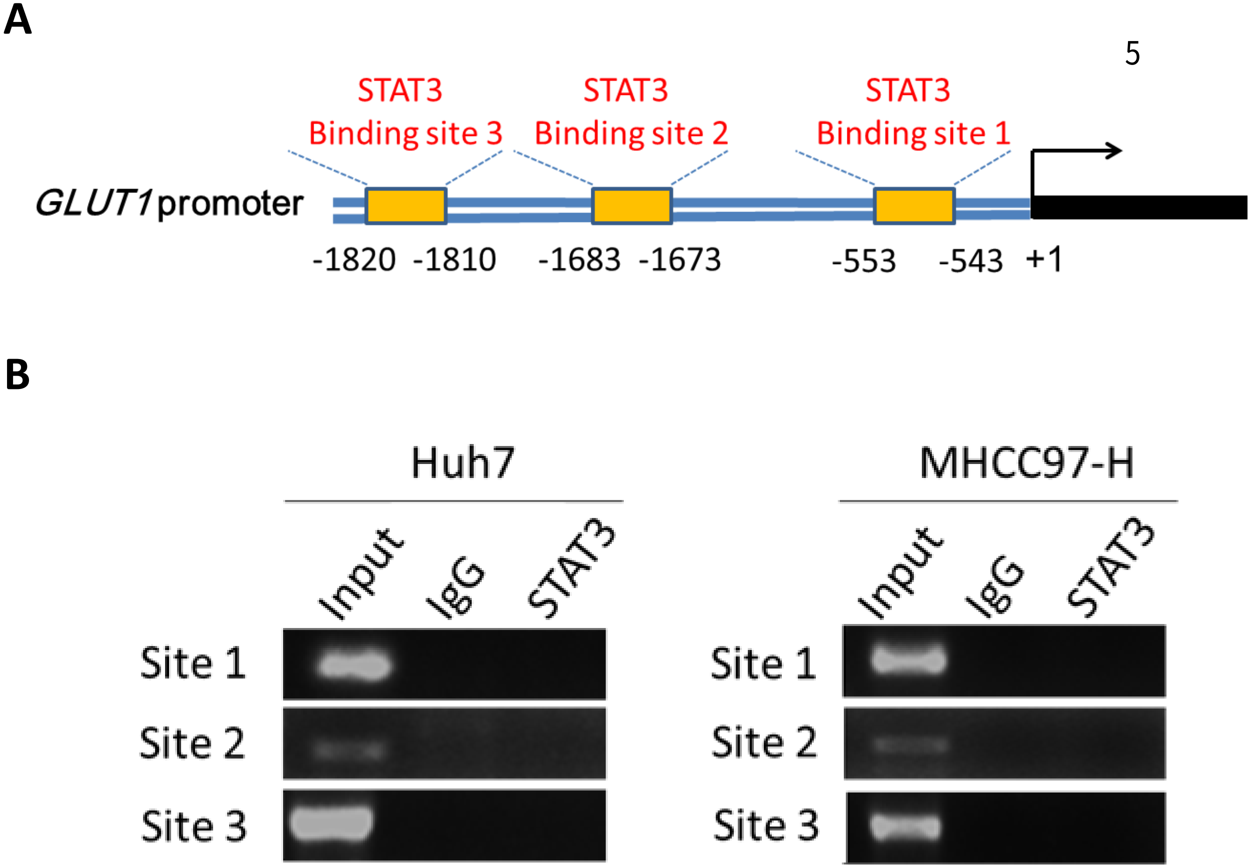

Fig. S4. STAT3 cannot bind to the GLUT1 promoter in HCC cells. (**A**) Schematic diagram of the putative STAT3 binding sites in the GLUT1 promoter. (**B**) ChIP analysis of the GLUT1 promoter using antibodies against STAT3 or IgG in Huh7 and MHCC97-H cells.

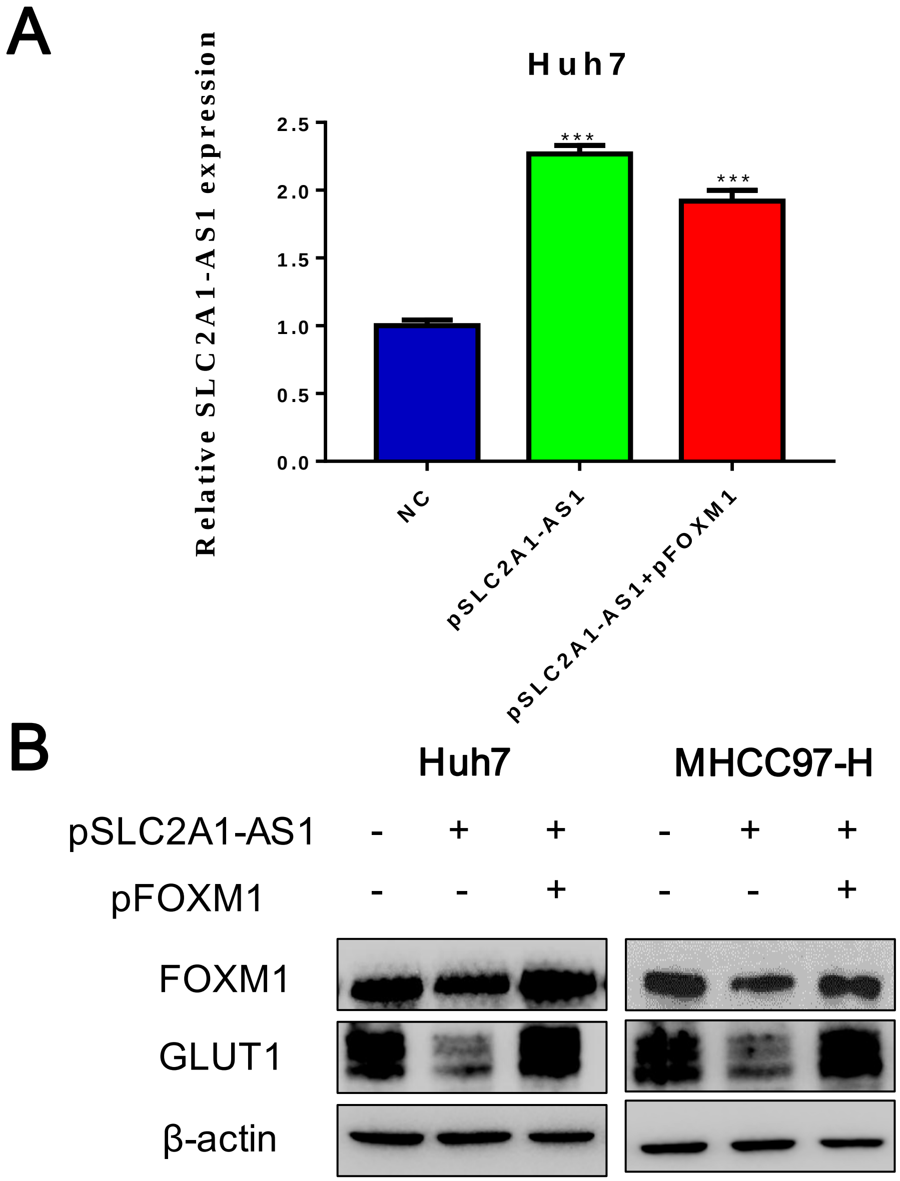

Fig. S5. FOXM1 is required for *SLC2A1-AS1*-mediated GLUT1 downregulation. HCC cells were transfected with control plasmids, *SLC2A1-AS1* overexpression plasmids or co-transfected with *SLC2A1-AS1* and FOXM1 overexpression plasmids, then the relative *SLC2A1-AS1* expression was assessed (**A**). (**B**) Relative expression of FOXM1 and GLUT1 was determined by western blot assays in Huh7 and MHCC97-H cells after transfection. ANOVA followed by post-hoc correction was performed to measure significance. ***P<0.001

Table S1. Primers used in qRT-PCR assays.

**Primer**	**Primer Sequence (5’-3’)**
SLC2A1-AS1-F	CTAAGATGAGAGGAGCTGGTAGG
SLC2A1-AS1-R	CAGAAGGAGTTTGCGTGGTT
GLUT1-F	GGCCAAGAGTGTGCTAAAGAA
GLUT1-R	ACAGCGTTGATGCCAGACAG
STAT3-F	GGGAGAGAGTTACAGGTTGGACAT
STAT3-R	AGACGCCATTACAAGTGCCA
FOXM1-F	GGGCGCACGGCGGAAGATGAA
FOXM1-R	CCACTCTTCCAAGGGAGGGCTC
β-actin-F	TAGTTGCGTTACACCCTTTCTTG
β-actin-R	TCACCTTCACCGTTCCAGTTT

Table S2. List of primary Antibodies.

Antibody	Catalog number	Company	Species	Dilution
GLUT1	PA5-16793	Thermo Fisher Scientific	Rabbit	1:1000
FOXM1	13147-1-AP	Proteintech	Rabbit	1:1000
STAT3	9319	Cell Signaling Technology	Mouse	1:1000
β-actin	3700	Cell Signaling Technology	Mouse	1:3000

Table S3. PCR primer sequences of DNA fragments targeted FOXM1 and GLUT1 promoters.

**Primer**	**Primer Sequence (5’-3’)**
GLUT1-binding site 1-F	AAGGAGGATGAGGTGGTG
GLUT1-binding site 1-R	GAGTATGGAGCCCTGAAAC
GLUT1-binding site 2-F	AGTCAGTTAGCCTGCCTTTC
GLUT1-binding site 2-R	TCTTGCCCAGGGATTGAC
GLUT1-binding site 3-F	GGCTTGGCTCACAAACGG
GLUT1-binding site 3-R	GAGTAGCGGGTGGAAAGC
FOXM1- binding site 1-F	CACCATCAAGCCCTGTCA
FOXM1- binding site 1-R	AGAGCCTTAGCCATACCG
FOXM1- binding site 2-F	TGCCATTGTATCTTCAGGG
FOXM1- binding site 2-R	GGCACCATGAACCCTACCT
FOXM1- binding site 3-F	TGCCATTGTATCTTCAGGG
FOXM1- binding site 3-R	CTTTGAGGGCTGCGTATT

Table S4. Univariate and multivariate Cox regression analyses of recurrence-free survival in 33 patients with HCC.

Clinicopathological characteristics	Univariate analysis			Multivariate analysis		
	HR	95 % *CI*	*P*	HR	95 % *CI*	*P*
Gender (female versus male)	0.276	0.063-1.204	*0.087*			
Age (>50 versus ≤50)	1.104	0.464-2.630	*0.823*			
AFP (>20 versus ≤20)	2.001	0.803-4.985	*0.748*			
Tumor size (≥5 versus <5cm)	1.665	0.703-3.944	*0.247*			
Tumor nodules (multiple versus single)	1.323	0.388-4.510	*0.655*			
Tumor differentiation (low versus high)	1.134	0.378-3.406	*0.822*			
Liver cirrhosis (with versus without)	1.673	0.695-4.028	*0.251*			
Microvascular invasion (present versus absent)	2.994	1.092-8.206	*0.033**	5.183	1.703-15.775	*0.004**
SLC2A1-AS1 (high versus low)	0.359	0.134-0.960	*0.033**	0.195	0.064-0.596	*0.004**
GLUT1 (high versus low)	2.543	1.055-6.128	*0.038**	1.705	0.700-4.154	*0.240*

Statistical analyses were performed by the Cox test analysis; HR hazard ratio, *CI* confidence interval, AFP alphafetoprotein; *$P<0.05$ was considered significant.

Table S5. Summary of proteins interacting with SLC2A1-AS1 uniquely by mass spectrometric analysis.

Protein name	Coverage(%)	Binding Length	Mass
EF1A3	32.9	462	50140.6
TBA1B	33.26	451	50151.2
H14	11.42	219	21865
ALDR	10.76	316	35853.1
MARCS	10.24	332	31554.2
TXND5	6.019	432	47628.5
ZYX	5.42	572	61276.9
KPRP	3.8	579	64135.2
RS11	13.29	158	18430.6
MOT4	5.591	465	49468.9
RL10L	8.411	214	24518.6
BASI	5.974	385	42200.1
UGPA	5.709	508	56939.8
RS12	8.333	132	14514.8
RRBP1	1.844	1410	152471.8
SYNC	1.46	548	62942.4
PSB6	4.603	239	25357.5
1433B	8.943	246	28082.2
SET	3.448	302	34882.1
MCM2	1.217	904	101895.2
MAGB2	5.016	319	35276.8
ACTG	66.4	375	41792.5
K2C6A	34.04	564	60044.5
DYH1	0.3695	4330	493948.4
COPG2	1.952	871	97621.3
IF2GL	2.331	472	51228.2
CPT1A	1.811	773	88366.9
SPB4	3.077	390	44853.7
CAZA1	5.245	286	32922.5
RL29	9.434	159	17751.9
ETFA	5.405	333	35079.2
AATM	1.86	430	47517.3
TRY6	8.097	247	26557.9
F10A5	3.523	369	41377.4
RL8	4.28	257	28024.5
QCR1	2.5	480	52645.3
S10A7	10.89	101	11470.9
EMD	4.724	254	28993.5
CALL5	5.479	146	15892.4
PDIA4	1.705	645	72931.9
AMPL	2.312	519	56165.8
DSRAD	0.8972	1226	136065.3
IF4B	2.128	611	69150.4
EIF3C	0.8762	913	105343
RASEF	1.486	740	82878.3
SF01	2.347	639	68329.5
AHSA1	3.254	338	38274.1
PFKAP	1.02	784	85595.4
PIMT	3.965	227	24636.2

COPD	2.153	511	57209.9
TLN2	0.4327	2542	271610.4
RL15	7.843	204	24145.9
S61A1	2.311	476	52264.2
STAT3	1.429	770	88067.2
DDX3X	1.208	662	73242.8
GBB1	3.529	340	37376.6
